# The Need of Dental Instruction in Medical Schools

**Published:** 1897-08

**Authors:** Edward Branigan

**Affiliations:** Boston, Mass.


### The Need of Dental Instruction in Medical Schools.

BY DR. EDWARD BRANIGAN, BOSTON, MASS.

Abstract of Paper read before the Dental Section, American Medical Association, June, 1897.

There is great need of dental instruction in medical schools. Physicans should know more about the teeth than they now do, and when medical students are given a better knowledge of the teeth and the many disturbances they cause, then will medical science do a little more for humanity than it is now doing. In this paper I shall confine myself to the narration of a few of the more common instances of the mismanaged treatment of diseases caused by the teeth. Abcesses caused by pulpless teeth are still poulticed and opened on the face by medical men. I have seen many hundreds of these. Of course this does not effect a cure, and in the course of time it is bound to dawn upon the patient, if not upon the doctor, that dental assistance is needed.

People are still being stabbed in the face and neck because a third molar kicks up a fuss in trying to get up into the mouth where it belongs. The medical treatment for these simple abcesses is sometimes varied a little. The sufferer may be kept in bed for a time, with temperature and pulse watched and medicine changed at intervals according to the symptoms. This sort of thing happens sometimes when the old tooth-root at the bottom of the trouble is so loose that the owner could pull it out with two fingers. Pus from an abcessed tooth is frequently discharged into the antrum. The extraction of the tooth followed by proper treatment would rob the medical profession of many patients who are supposed to suffer from, and are treated for catarrh. Serious throat diseases are frequently caused by pulpless teeth and sometimes by unerupted or retained teeth. I find that not all throat specialists, even, are aware of this. A more intimate knowledge of other specialists regarding the teeth would be of great value, for instance, to aurists. Severe and painful inflammation of the ear, caused by decayed teeth, is common. The removal of a filling from a pulpless tooth has, under my observation, made a number of sufferers from aural treatment happy. Unerupted wisdom, and sometimes supernumerary, teeth, are the cause of many obscure ear troubles.

Oculists are now tracing a variety of diseases back to the teeth. Tumors of the nose may be caused by a dead tooth-pulp. Treatment of the tumor without a knowledge of the cause is not calculated to make the owner of the nose happy. The victims of the so-called neuralgia who, after weeks, months, and even years of anti-neuralgic drugging, die or find relief at the hands of some one who knows something about the teeth, are countless. Thousands of people to-day are being drugged for neuralgia who are really suffering from some form of dental irritation. The authorities in charge of the management of hospitals should know more about dentistry, and the medical directors of public institutions can find a vast amount of good work ready for them when they realize the importance of having dental service given to those in their charge.

